# ESCRT machinery plays a role in microautophagy in yeast

**DOI:** 10.1186/s12860-020-00314-w

**Published:** 2020-10-07

**Authors:** Shamsul Morshed, Most Naoshia Tasnin, Takashi Ushimaru

**Affiliations:** 1 Graduate School of Science and Technology, Shizuoka University, Ohya 836, Suruga-ku, Shizuoka, 422-8021 Japan; 2 Department of Science, Shizuoka University, Ohya 836, Suruga-ku, Shizuoka, 422-8021 Japan

**Keywords:** AP-3 pathway, ESCRT, Microautophagy, Pho8, Vph1, VPS pathway

## Abstract

**Background:**

Microautophagy, which degrades cargos by direct lysosomal/vacuolar engulfment of cytoplasmic cargos, is promoted after nutrient starvation and the inactivation of target of rapamycin complex 1 (TORC1) protein kinase. In budding yeast, microautophagy has been commonly assessed using processing assays with green fluorescent protein (GFP)-tagged vacuolar membrane proteins, such as Vph1 and Pho8. The endosomal sorting complex required for transport (ESCRT) system is proposed to be required for microautophagy, because degradation of vacuolar membrane protein Vph1 was compromised in ESCRT-defective mutants. However, ESCRT is also critical for the vacuolar sorting of most vacuolar proteins, and hence reexamination of the involvement of ESCRT in microautophagic processes is required.

**Results:**

Here, we show that the Vph1-GFP processing assay is unsuitable for estimating the involvement of ESCRT in microautophagy, because Vph1-GFP accumulated highly in the prevacuolar class E compartment in ESCRT mutants. In contrast, GFP-Pho8 and Sna4-GFP destined for vacuolar membranes via an alternative adaptor protein-3 (AP-3) pathway, were properly localized on vacuolar membranes in ESCRT-deficient cells. Nevertheless, microautophagic degradation of GFP-Pho8 and Sna4-GFP after TORC1 inactivation was hindered in ESCRT mutants, indicating that ESCRT is indeed required for microautophagy after nutrient starvation and TORC1 inactivation.

**Conclusions:**

These findings provide evidence for the direct role of ESCRT in microautophagy induction.

## Background

In microautophagy, cytoplasmic cargos are directly engulfed by lysosomal/vacuolar membranes, sorted into the vacuolar lumen, and degraded [1–3]. Because vacuolar membrane proteins together with vacuolar membranes are degraded in the vacuole in the course of microautophagy, overall microautophagic flux is estimated using green fluorescent protein (GFP)-tagged vacuolar transmembrane proteins, Vph1 (a subunit of the vacuolar-ATPase V0 domain) and Pho8 (vacuolar alkaline phosphatase) in the budding yeast *Saccharomyces cerevisiae* [4]. When Vph1-GFP and GFP-Pho8 are incorporated into the vacuolar lumen by microautophagy, Vph1 and Pho8, but not the stable GFP moiety, are degraded by vacuolar proteases, generating free GFP, which is detectable by immunoblotting. Nutrient starvation or inactivation of target of rapamycin complex 1 (TORC1) protein kinase evokes microautophagy [4–6].

The endosomal sorting complex required for transport (ESCRT) system was originally identified as being involved in the formation of intraluminal vesicles within multivesicular bodies [7–9]. The ESCRT-0 complex binds to ubiquitinated proteins on endosomal membranes and recruits the ESCRT-I and -II complexes, eliciting the assembly of ESCRT-III, which promotes the invagination, constriction, and abscission of endosomal membranes. In microautophagy, carbon starvation after diauxic shift or TORC1 inactivation promotes recruitment of ESCRT-0 onto vacuolar membranes [4, 10]. Furthermore, free GFP production from Vph1-GFP after diauxic shift was significantly impeded in cells lacking ESCRT-0, −I, −II or -III, inferring a model where ESCRT is required for the deformation of the vacuolar membrane and, thus, also microautophagy [4]. However, most proteins destined for vacuoles pass through late endosomes, named the vacuolar protein sorting (VPS) pathway (or the carboxypeptidase Y pathway), and hence they are trapped in the class E compartment in ESCRT mutants [11, 12]. This suggested a possibility that Vph1-GFP is not located properly on vacuolar membranes during microautophagy induction, thereby causing a reduction in the autophagic degradation of Vph1-GFP. Thus, it remains unclear whether or not ESCRT is directly necessary for microautophagic processes on vacuolar membranes.

We found that Vph1 accumulated in the perivacuolar class E compartment in *vps27∆* cells defective in ESCRT-0, but we did not further investigate the involvement of ESCRT in sorting of Vph1-GFP and GFP-Pho8 [5]. In addition, we also found that loss of ESCRT compromised microautophagic degradation of GFP-Pho8 after rapamycin treatment, but we did not check whether Pho8 was properly localized on vacuolar membranes in ESCRT mutant cells [10]. Here, we show that Pho8 destined for vacuolar membranes via an alternative adaptor protein-3 (AP-3) pathway, but not Vph1, is correctly targeted to vacuolar membranes even in ESCRT mutants and after TORC1 inactivation. Nevertheless, free GFP production from GFP-Pho8 after TORC1 inactivation was hindered in ESCRT mutants. These findings demonstrate that the ESCRT machinery is genuinely necessary for microautophagic processes. Another AP-3 pathway-dependent vacuolar membrane protein Sna4 confirmed this idea.

## Results

### Vph1-GFP is an unsuitable tool to assess the involvement of ESCRT in microautophagy

**Fig. 1:**
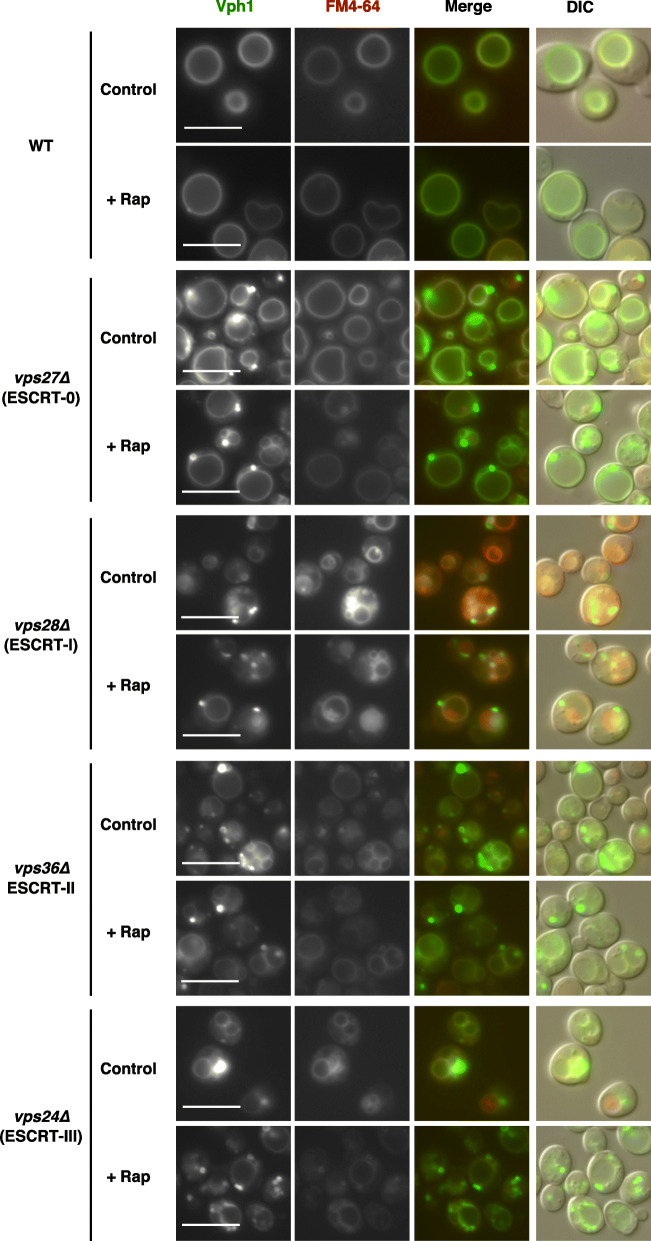
Vph1-GFP is not properly localized on vacuolar membranes in ESCRT mutants. Exponentially growing cells of strains SCU2684 (wild-type; BY4741), SCU5456 (*vps27∆*), SCU6187 (*vps28∆*), SCU6188 (*vps36∆*), and SCU4337 (*vps24∆*) harboring plasmid pSCU2425 (pVPH1-GFP) pretreated with FM4–64 (10 ng/ml) were treated with 200 ng/ml rapamycin for 6 h. Fluorescence micrographs are shown. Scale bars, 5 μm

To assess microautophagic flux/activity using a processing assay with a GFP-tagged vacuolar membrane protein, its proper localization in vacuolar membranes is prerequisite. First, we assessed whether Vph1 is correctly localized in cells defective in each ESCRT complex, ESCRT-0 to -III, both in normal (nutrient-rich and TORC1 active) conditions and during microautophagy induction after TORC1 inactivation. We found that ESCRT-lacking mutant cells, with mutations in Vps27 (ESCRT-0), Vps28 (ESCRT-I), Vps36 (ESCRT-II) or Vps24 (ESCRT-III), showed massive accumulation of Vph1-GFP in the perivacuolar class E compartment in normal (nutrient-rich and TORC1 active) conditions (Fig. 1, control) as described previously [5, 13–16]. This clearly confirmed that Vph1 is delivered to the vacuolar membrane via the VPS pathway. The aberrant accumulation of Vph1-GFP still remained during microautophagy induction after rapamycin treatment (Fig. 1, +Rap). Namely, Vph1-GFP did not properly reach the vacuolar surface in ESCRT-deficient cells regardless of TORC1 activity. This demonstrated that the Vph1-GFP processing assay is not suitable to evaluate whether ESCRT is directly implicated in microautophagic processes, although Oku et al. proposed it based on results obtained using this assay [4].

### GFP-Pho8 is a suitable marker to evaluate the involvement of ESCRT in microautophagy

**Fig. 2:**
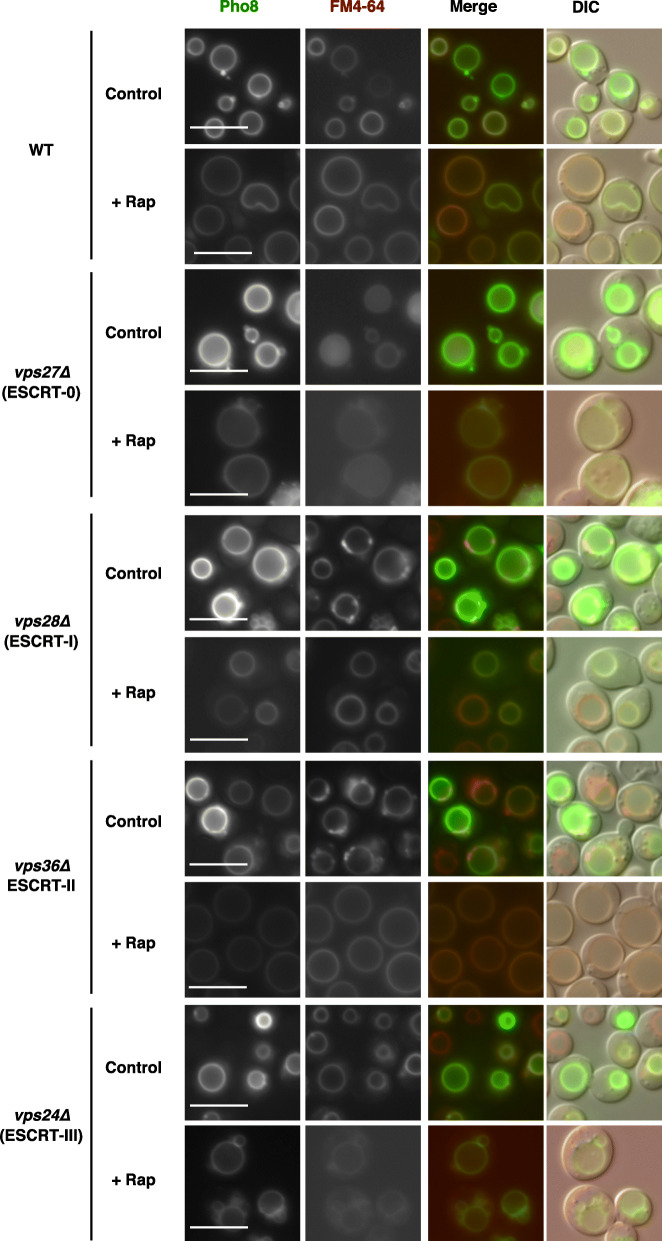
GFP-Pho8 is properly localized on vacuolar membranes in ESCRT mutants. Cells of strains SCU2684 (wild-type; BY4741), SCU5456 (*vps27∆*), SCU6187 (*vps28∆*), SCU6188 (*vps36∆*), and SCU4337 (*vps24∆*) harboring plasmid pSCU2366 (pGFP-PHO8) pretreated with FM4–64 were treated with rapamycin for 6 h. Fluorescence micrographs are shown. Scale bars, 5 μm

Next, we similarly assessed GFP-Pho8. In contrast to most vacuolar proteins trafficked via the VPS pathway, a small number of proteins including the vacuolar membrane alkaline phosphatase (ALP) Pho8 are transported directly from the Golgi to the vacuolar surface, even in class E mutants, via the adaptor protein-3 (AP-3) pathway (or the ALP pathway) [14, 17–19]. We also observed that GFP-Pho8 was appropriately located on vacuolar membranes in ESCRT mutants in normal conditions (Fig. 2, control). Furthermore, its vacuolar localization was not lost during microautophagy induction after cells were treated with rapamycin (Fig. 2, +Rap). Thus, GFP-Pho8 is properly localized on the vacuolar membrane in ESCRT mutants regardless of TORC1 activity. We concluded that GFP-Pho8 is a suitable marker to address the involvement of ESCRT in microautophagy.

### ESCRT is required for microautophagy after TORC1 inactivation

**Fig. 3:**
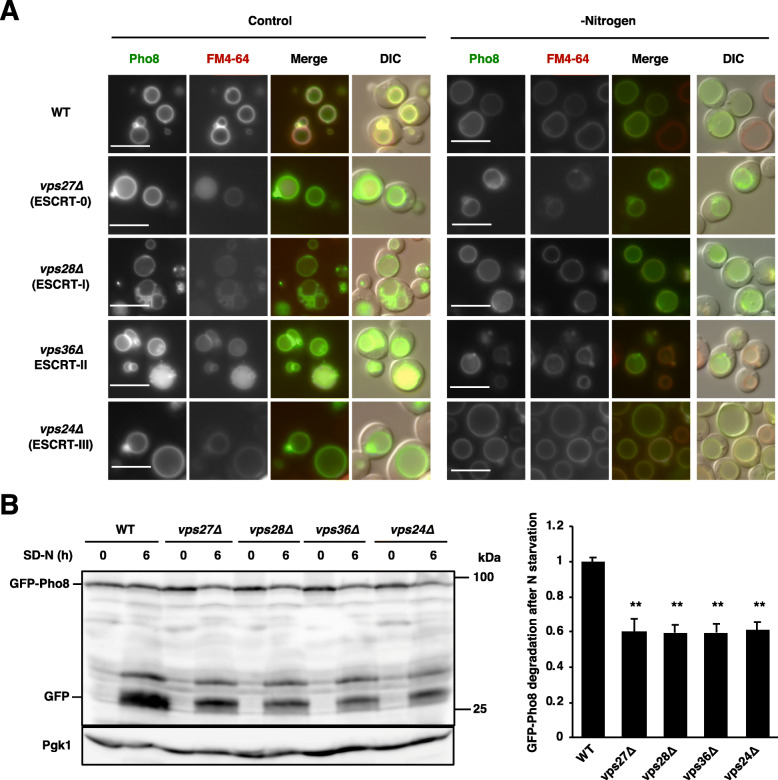
ESCRT is required for microautophagy after nitrogen starvation. **a** Cells of strains SCU2684 (wild-type; BY4741), SCU5456 (*vps27∆*), SCU6187 (*vps28∆*), SCU6188 (*vps36∆*), and SCU4337 (*vps24∆*) harboring plasmid pSCU2366 (pGFP-PHO8) pretreated with FM4–64 were transferred to nitrogen-depleted medium (SD-N) for 6 h. Fluorescence micrographs are shown. Scale bars, 5 μm. **b** The same strains as used in panel **a** was similarly treated with nitrogen starvation. Whole cell extracts were subjected to western blotting using an anti-GFP antibody. Pgk1 was detected as a loading control using an anti-Pgk1 antibody. Free GFP processed from GFP-Pho8 nitrogen starvation was measured, and quantified by calculating the ratio of cleaved free GFP versus the sum of uncleaved GFP-Pho8 and free GFP. The average (± standard deviation) was determined from three independent experiments, and relative values were normalized against the value in control cells are shown. For group comparisons, *p*-values were calculated using two-way ANOVA with Bonferroni correction. **, *p* < 0.001

In a parallel study, we recently reported that free GFP generation from GFP-Pho8 after treatment with rapamycin was massively compromised in cells defective in ESCRT, *vps27∆*, *vps28∆*, *vps36∆*, and *vps24∆* cells [10]. Given the proper localization of GFP-Pho8 on vacuolar membranes in these ESCRT mutants, these observations indicated that ESCRT is required for microautophagy itself. To further confirm this we subjected cells to nitrogen starvation, which is a natural condition where TORC1 is inactivated. GFP-Pho8 still showed proper distribution on the vacuolar surface after nitrogen starvation even in ESCRT mutants (Fig. 3a). Autophagic degradation of GFP-Pho8 after nitrogen starvation was mildly reduced in these ESCRT mutants (Fig. 3b) (see “Discussion”). These findings confirmed the idea that ESCRT is indeed required for proper microautophagic induction after TORC1 inactivation.

### Search for other appropriate markers of microautophagy

**Fig. 4:**
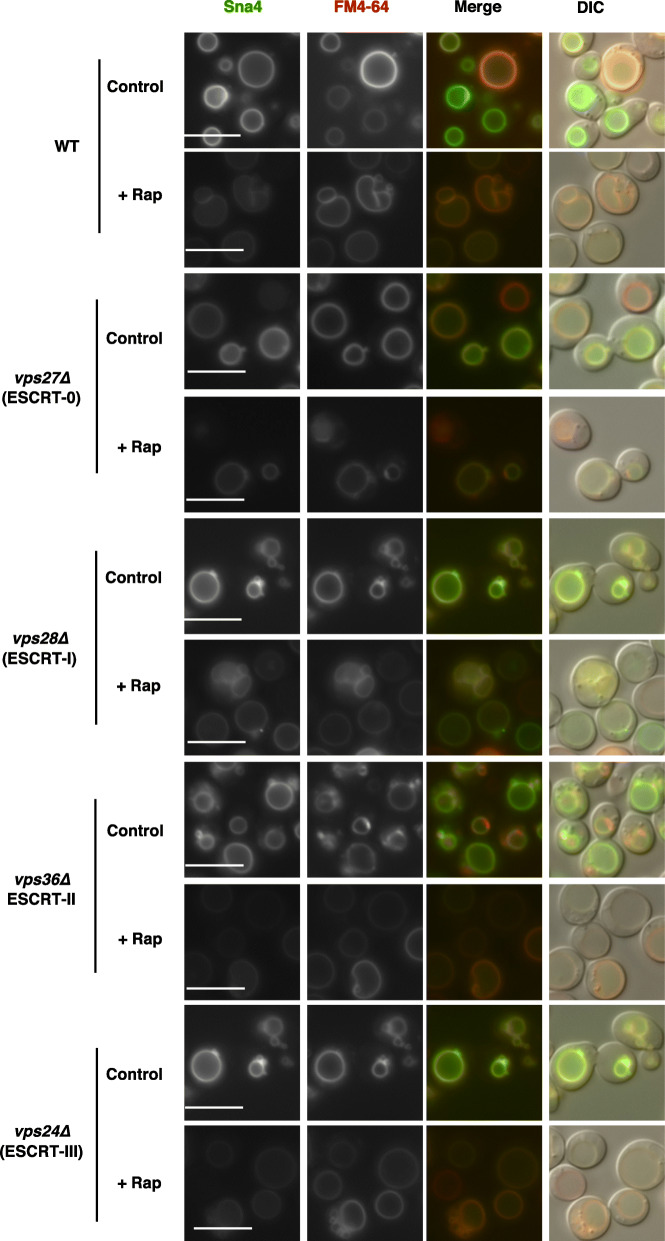
Localization of Sna4-GFP on the vacuolar membrane in ESCRT mutants. Cells of strains SCU2684 (wild-type; BY4741), SCU5456 (*vps27∆*), SCU6187 (*vps28∆*), SCU6188 (*vps36∆*), and SCU4337 (*vps24∆*) harboring plasmid pSCU2475 (pSNA4-GFP) pretreated with FM4–64 were treated with rapamycin for 6 h. Fluorescence micrographs are shown. Scale bars, 5 μm

**Fig. 5:**
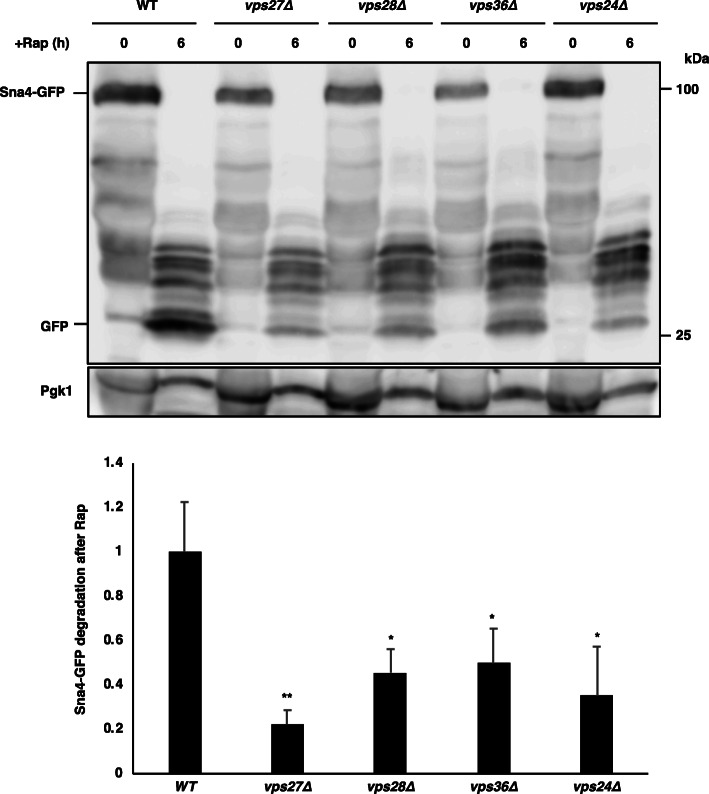
Detection of degradation products of Sna4-GFP. Cells of strains SCU2684 (wild-type; BY4741), SCU5456 (*vps27∆*), SCU6187 (*vps28∆*), SCU6188 (*vps36∆*), and SCU4337 (*vps24∆*) harboring plasmid pSCU2475 (pSNA4-GFP) were treated with rapamycin for 6 h. Whole cell extracts were subjected to western blotting using the anti-GFP antibody. To assess microautophagic flux, the ratios of cleaved free GFP versus Pgk1 were calculated and the values relative to that in wild-type cells are shown. The average and standard deviation were determined from three independent experiments, and relative values were normalized against the value in control cells are shown. *p*-values were calculated using two-way ANOVA with Bonferroni correction. *, *p* < 0.05; **, *p* < 0.005

Thus, proteins destined for vacuolar membranes via the AP-3 pathway (the ALP pathway) have potential as appropriate markers to investigate ESCRT-mediated microautophagy. We assessed this using the following vacuolar membrane proteins targeted via the AP-3 pathway: Nyv1 (vacuolar v-SNARE), Yck3 (casein kinase) and Sna4 (protein of unknown function) [20–22]. We found that Nyv1-GFP was not clearly distributed on vacuolar membranes even in wild-type cells (Additional file 1: Fig. S1a). By contrast, clear localization of Yck3-GFP on vacuolar membranes was found in wild-type cells in the absence or presence of rapamycin (Additional file 1: Figs. S1a, b). However, unlike Vph1-GFP and GFP-Pho8, Yck3-GFP did not generate free GFP after rapamycin treatment, although Yck3-GFP was almost lost (Additional file 1: Fig. S1c). This suggested that Yck3 is degraded outside vacuoles (see “Discussion”). Thus, Nyv1-GFP and Yck3-GFP were not suitable for the assessment microautophagic flux. Finally, we found that Sna4-GFP was located on vacuolar membranes in wild-type cells in the absence and presence of rapamycin and that similar vacuolar localization was maintained in ESCRT mutants, although Sna4-GFP signals on the vacuolar membranes were largely lost after rapamycin treatment (Fig. 4, Additional file 1: Fig. S2). Sna4-GFP produced free GFP in wild-type cells after rapamycin treatment (Fig. 5, see also Additional file 1: Fig. S3), which was repressed in *vps27∆* cells, similar to GFP-Pho8 [10]. In addition, free GFP generation from Sna4-GFP after rapamycin treatment was also reduced in *vps28∆*, *vps36∆*, and *vps24∆* cells, although uncharacterized protein bands accumulated additionally in wild-type and the mutant cells. These findings confirmed the hypothesis that ESCRT is required for proper microautophagy induction.

## Discussion

In this study, we showed that GFP-Pho8 and Sna4-GFP are suitable tools to evaluate the involvement of ESCRT in microautophagic processes on vacuolar membranes and that ESCRT is genuinely required for this process after TORC1 inactivation. In contrast, Vph1-GFP is not suitable for use in this assay, because Vph1-GFP was not correctly localized on the vacuolar membranes in ESCRT mutants.

We tested whether other vacuolar membrane proteins sorted via the AP-3 pathway (Nyv1 and Yck3) are suitable as markers of microautophagic flux. However, they were found not to be useable markers for microautophagy assays. In the course of this study, we noticed that the vacuolar membrane protein Yck3 was not degraded in the vacuole after TORC1 inactivation, although microautophagy is induced: there was no accumulation of free GFP (a marker of vacuolar degradation) from Yck3-GFP after rapamycin treatment, although Yck3-GFP was lost. It is plausible that Yck3 is degraded by proteasomes in the cytoplasm after TORC1 inactivation. The armadillo repeat protein Vac8 is localized on the vacuolar membrane, and has multiple functions including vacuole inheritance [23, 24]. No free GFP production from Vac8-GFP appeared after rapamycin treatment (Morshed et al., unpublished data). These findings indicated that some, but not all, vacuolar membrane proteins are degraded by microautophagy after TORC1 inactivation. It is an interesting open question how some vacuolar membrane proteins escape from microautophagic degradation after TORC1 inactivation. Furthermore, Sna4-GFP was lost with no clear free GFP accumulation after rapamycin treatment in *vps27∆* cells (Fig. 5), indicating that Sna4 was degraded outside vacuoles in specific situations. These findings imply that some vacuolar membrane proteins are degraded by multiple pathways after TORC1 inactivation.

In the case of nitrogen starvation, loss of ESCRT mildly reduced nitrogen starvation-induced microautophagy (Fig. 3b). This suggested a possibility that ESCRT-independent microautophagy is promoted after nitrogen starvation. Alternatively, a microautophagy-independent pathway might mediate degradation of vacuolar membrane proteins under these conditions. It has been reported that ESCRT-independent homotypic vacuole–vacuole fusion mediates degradation of vacuolar membrane proteins (the intraluminal fragment pathway) [25].

This study concluded that ESCRT is indeed involved in microautophagic processes in budding yeast. However, the molecular mechanism of ESCRT-mediated microautophagy is still largely unknown. This study provides a caution for the selection of markers to assess microautophagic flux. To evaluate microautophagic activity in mutants using GFP-tagged vacuolar membrane proteins, confirmation of their proper localization on vacuolar membranes is prerequisite. It is suggested that microautophagy is involved in protein homeostasis in mammalian cells [26]. However, the molecular mode and physiological roles of mammalian microautophagy are still largely unknown [27, 28]. This study is helpful for dissection of the mechanism and physiological meaning of microautophagy not only in budding yeast but also other organisms including humans.

## Conclusions

Microautophagic degradation of GFP-Pho8 and Sna4-GFP after TORC1 inactivation was hindered in ESCRT-deficient cells, although they were properly sorted onto vacuolar membranes. This fact indicates that ESCRT is indeed required for microautophagy after nutrient starvation and TORC1 inactivation.

## Methods

### Strains and media

The *S. cerevisiae* strains and plasmids are listed in Additional files: Tables S1 and S2, respectively. Glucose-containing YPAD (YPD containing 0.01% adenine) and synthetic minimal medium (SD) complemented with the appropriate nutrients for plasmid maintenance were prepared using standard methods. For assessment of microautophagy after rapamycin treatment, when cells harbored plasmids, cells were precultured in SD medium with the appropriate nutrients, and then cultured in YPAD, followed by rapamycin treatment. For nitrogen-starvation experiments, cells were transferred into SD-N medium without ammonium sulfate.

### Western blotting analysis

Exponentially growing cells were used and proteins were extracted using a post-alkaline extraction method in accordance with a previous report [29]. We used the following antibodies: anti-GFP mouse monoclonal antibody (Santa Cruz, Cat#sc-9996; RRID: AB_627695) and anti-Pgk1 mouse monoclonal antibody (Abcam, Cat# ab113687; RRID: AB_10861977). All western blotting experiments were performed independently at least three times to confirm the reproducibility of the results. Relative protein amounts were measured using ImageJ software. To assess microautophagic flux, free GFP processed from of GFP-Pho8 after rapamycin treatment or nitrogen starvation was measured and quantified by calculating the ratio of cleaved free GFP versus the sum of uncleaved GFP-fused proteins and free GFP, and the values relative to that in wild-type cells are shown, as described previously [30]. In the case of Sna4-GFP, uncleaved Sna4-GFP was almost lost after rapamycin treatment. Therefore, to assess microautophagic flux using Sna4-GFP, the ratios of cleaved free GFP versus Pgk1 were calculated, and the values relative to that in wild-type cells are shown. The average and standard deviation were determined for each sample from three independent experiments, and relative values normalized against the value in control cells are shown. For group comparison, data analysis was performed with the software GraphPad Prism (v. 8.1.2) followed by two-way ANOVA (or mixed model) with Bonferroni correction for multiple comparisons in statistical hypothesis testing.

### Microscope observations

Exponentially growing cells were used for experiments. Cells were stained with the vacuolar membrane dye FM4–64 (10 ng/ml) for 1 h, washed to remove free dye and further incubated at least for 1.5 h prior to treatment with rapamycin or nitrogen starvation. Cell, GFP, and FM4–64 images were captured using a Carl Zeiss Axio Imager M1 microscope with a cooled CCD camera (Carl Zeiss AxioCam MRm) [10]. All microscope observations were performed independently at least three times to confirm the reproducibility of the results. For quantification of intensities of Sna4-GFP signals on the vacuolar membrane, we used a microscopy-equipped software (Carl Zeiss AxioVision) according to a previous study [31].

### Statistical analysis

Statistical analysis was performed using Microsoft Excel and GraphPad Prism 8 software.

## Supplementary information

**Additional file 1 Fig. S1.** Characterizations of Yck3 and Nyv1, vacuolar membrane proteins sorted via the AP-3 pathway. **a** Fluorescence micrographs of cells of strains SCU6206 (*YCK3-GFP*) and SCU6207 (*NYV1-GFP*). Fluorescence micrographs of cells are shown. Scale bars, 5 μm. **b** Cells of strains SCU6206 (*YCK3-GFP*) were treated with rapamycin for 6 h. Scale bars, 5 μm. **c** Cells of strain SCU6206 (*YCK3-GFP*) were treated with rapamycin for 6 h. Whole cell extracts were subjected to western blotting using the anti-GFP antibody. **Fig. S2.** Intensity of Sna4-GFP signals on the vacuolar membranes (Related to Fig. 4). **a** Illustration for measurement of intensities of fluorescent signals of Sna4-GFP on vacuolar membranes. **b** Examples for measurement of the intensities of GFP on the vacuolar membrane using a captured cell images. Cells expressing a GFP-tagged vacuolar membrane protein were treated with rapamycin for 6 h. **c** The intensities of Cells of strains SCU2684 (wild-type; BY4741), SCU5456 (*vps27∆*), SCU6187 (*vps28∆*), SCU6188 (*vps36∆*), and SCU4337 (*vps24∆*) harboring plasmid pSCU2475 (pSNA4-GFP) signals on vacuolar membranes in non-treated control and rapamycin-treated cells are shown in the box plot with dots. Sample sizes are 15. *p*-values were calculated using two-way ANOVA test (or mixed model) with Bonferroni correction. ***, *p* < 0.0001. *p* values between intensities in wild-type and each ESCRT mutant were higher than 0.05 before and after rapamycin treatment. **Fig. S3.** Sna4-GFP signals before and after rapamycin treatment (Related to Fig. 5). Cells of strain SCU2684 (wild-type) harboring plasmid pSCU2475 (pSNA4-GFP) were treated with rapamycin for 6 h. Whole cell extracts were subjected to western blotting using anti-GFP antibody. For the control, wild-type cells without pSNA4-GFP were used. **Fig. S4.** Independent western blot images with quantifications (Related to Fig. 3**b**). **Fig. S5.** Independent western blot images with quantifications (Related to Fig. 5).

**Additional file 2 Table S1.** Yeast strains used in this study.

**Additional file 3 Table S2.** Plasmids used in this study.

## Data Availability

Data and materials used in the current study are available from the corresponding authors on request.

## Abbreviations

ALP: Alkaline phosphatase
AP-3: Adaptor protein-3
ESCRT: Endosomal sorting complex required for transport
GFP: Green fluorescent protein
TORC1: Target of rapamycin complex 1
VPS: Vacuolar protein sorting
