# Supplementary material for: ESCRT machinery plays a role in microautophagy in yeast
Source: BMC Mol Cell Biol. 2020 Oct 7;21:70. doi: 10.1186/s12860-020-00314-w (PMC7542719; doi:10.1186/s12860-020-00314-w)
Supplement: Supplementary file 1 — Additional file 1 Fig. S1. Characterizations of Yck3 and Nyv1, vacuolar membrane proteins sorted via the AP-3 pathway. a Fluorescence micrographs of cells of strains SCU6206 (YCK3-GFP) and SCU6207 (NYV1-GFP). Fluorescence micrographs of cells are shown. Scale bars, 5 μm. b Cells of strains SCU6206 (YCK3-GFP) were treated with rapamycin for 6 h. Scale bars, 5 μm. c Cells of strain SCU6206 (YCK3-GFP) were treated with rapamycin for 6 h. Whole cell extracts were subjected to western blotting using the anti-GFP antibody. Fig. S2. Intensity of Sna4-GFP signals on the vacuolar membranes (Related to Fig. 4). a Illustration for measurement of intensities of fluorescent signals of Sna4-GFP on vacuolar membranes. b Examples for measurement of the intensities of GFP on the vacuolar membrane using a captured cell images. Cells expressing a GFP-tagged vacuolar membrane protein were treated with rapamycin for 6 h. c The intensities of Cells of strains SCU2684 (wild-type; BY4741), SCU5456 (vps27∆), SCU6187 (vps28∆), SCU6188 (vps36∆), and SCU4337 (vps24∆) harboring plasmid pSCU2475 (pSNA4-GFP) signals on vacuolar membranes in non-treated control and rapamycin-treated cells are shown in the box plot with dots. Sample sizes are 15. p-values were calculated using two-way ANOVA test (or mixed model) with Bonferroni correction. ***, p < 0.0001. p values between intensities in wild-type and each ESCRT mutant were higher than 0.05 before and after rapamycin treatment. Fig. S3. Sna4-GFP signals before and after rapamycin treatment (Related to Fig. 5). Cells of strain SCU2684 (wild-type) harboring plasmid pSCU2475 (pSNA4-GFP) were treated with rapamycin for 6 h. Whole cell extracts were subjected to western blotting using anti-GFP antibody. For the control, wild-type cells without pSNA4-GFP were used. Fig. S4. Independent western blot images with quantifications (Related to Fig. 3b). Fig. S5. Independent western blot images with quantifications (Related to Fig. 5). [file 12860_2020_314_MOESM1_ESM.pdf]

Additional file 1: Fig. S1–S5

Title:

ESCRT machinery plays a role in microautophagy in yeast

Author names:

Shamsul Morshed, Most Naoshia Tasnin and Takashi Ushimaru

Fig. S1

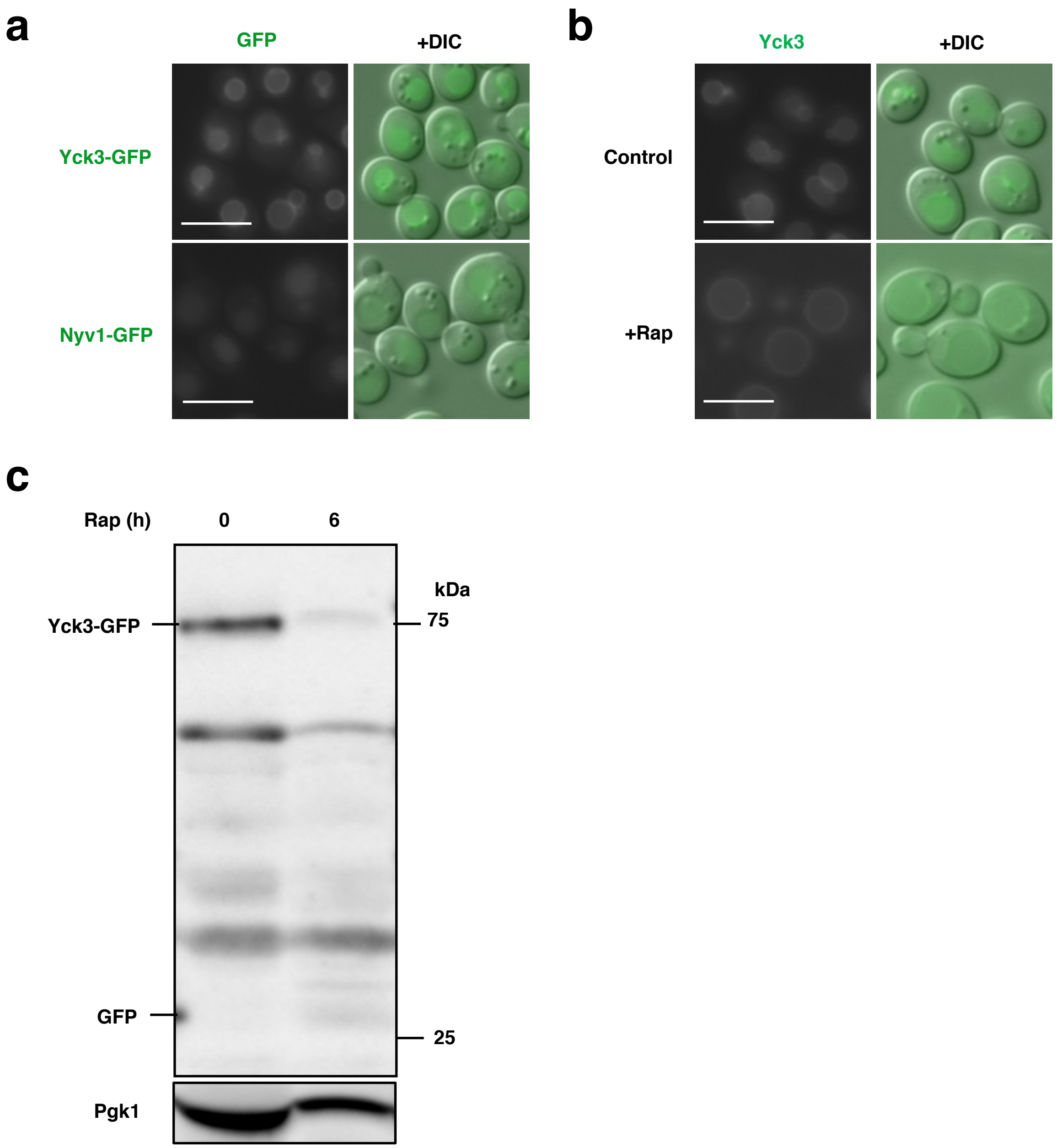

Fig. S1. Characterizations of Yck3 and Nyv1, vacuolar membrane proteins sorted via the AP-3 pathway.

(a) Fluorescence micrographs of cells of strains SCU6206 (*YCK3-GFP*) and SCU6207 (*NYV1-GFP*). Fluorescence micrographs of cells are shown. Scale bars, 5 μm. (b) Cells of strains SCU6206 (*YCK3-GFP*) were treated with rapamycin for 6 h. Scale bars, 5 μm. (c) Cells of strain SCU6206 (*YCK3-GFP*) were treated with rapamycin for 6 h. Whole cell extracts were subjected to western blotting using the anti-GFP antibody.

Fig. S2

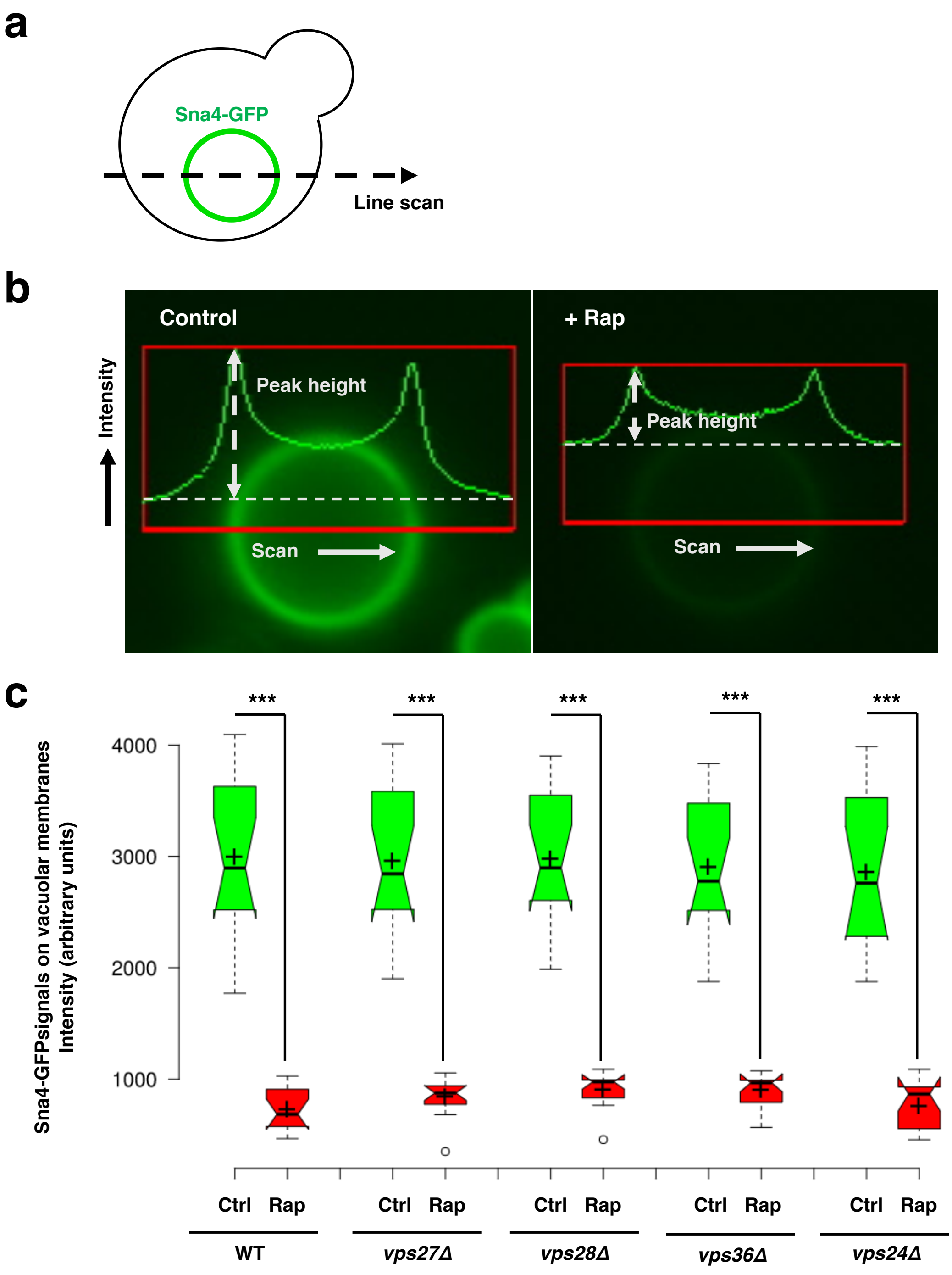

Fig. S2. Intensity of Sna4-GFP signals on the vacuolar membranes (Related to Figure 4). (a) Illustration for measurement of intensities of fluorescent signals of Sna4-GFP on vacuolar membranes. (b) Examples for measurement of the intensities of GFP on the vacuolar membrane using a captured cell images. Cells expressing a GFP-tagged vacuolar membrane protein were treated with rapamycin for 6 h. (c) The intensities of Cells of strains SCU2684 (wild-type; BY4741), SCU5456 (*vps27Δ*), SCU6187 (*vps28Δ*), SCU6188 (*vps36Δ*), and SCU4337 (*vps24Δ*) harboring plasmid pSCU2475 (pSNA4-GFP) signals on vacuolar membranes in non-treated control and rapamycin-treated cells are shown in the box plot with dots. Sample sizes are 15. *p*-values were calculated using two-way ANOVA test (or mixed model) with Bonferroni correction. ***, $p < 0.0001$. *p* values between intensities in wild-type and each ESCRT mutant were higher than 0.05 before and after rapamycin treatment.

Fig. S3

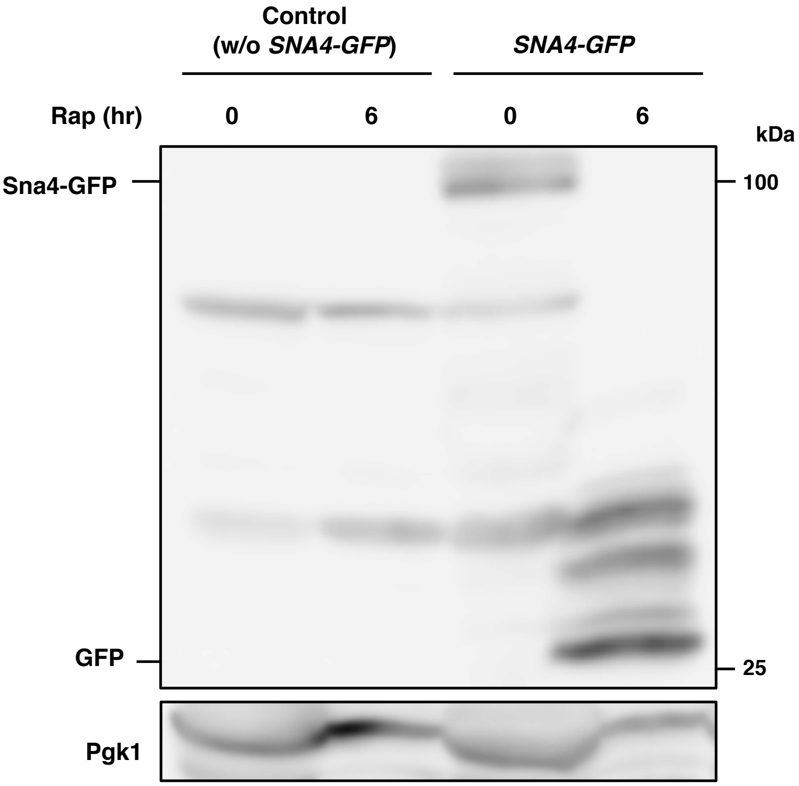

Fig. S3. Sna4-GFP signals before and after rapamycin treatment (Related to Figure 5). Cells of strain SCU2684 (wild-type) harboring plasmid pSCU2475 (pSNA4-GFP) were treated with rapamycin for 6 h. Whole cell extracts were subjected to western blotting using the anti-GFP antibody. For the control, wild-type cells without pSNA4-GFP were used.

Fig. S4

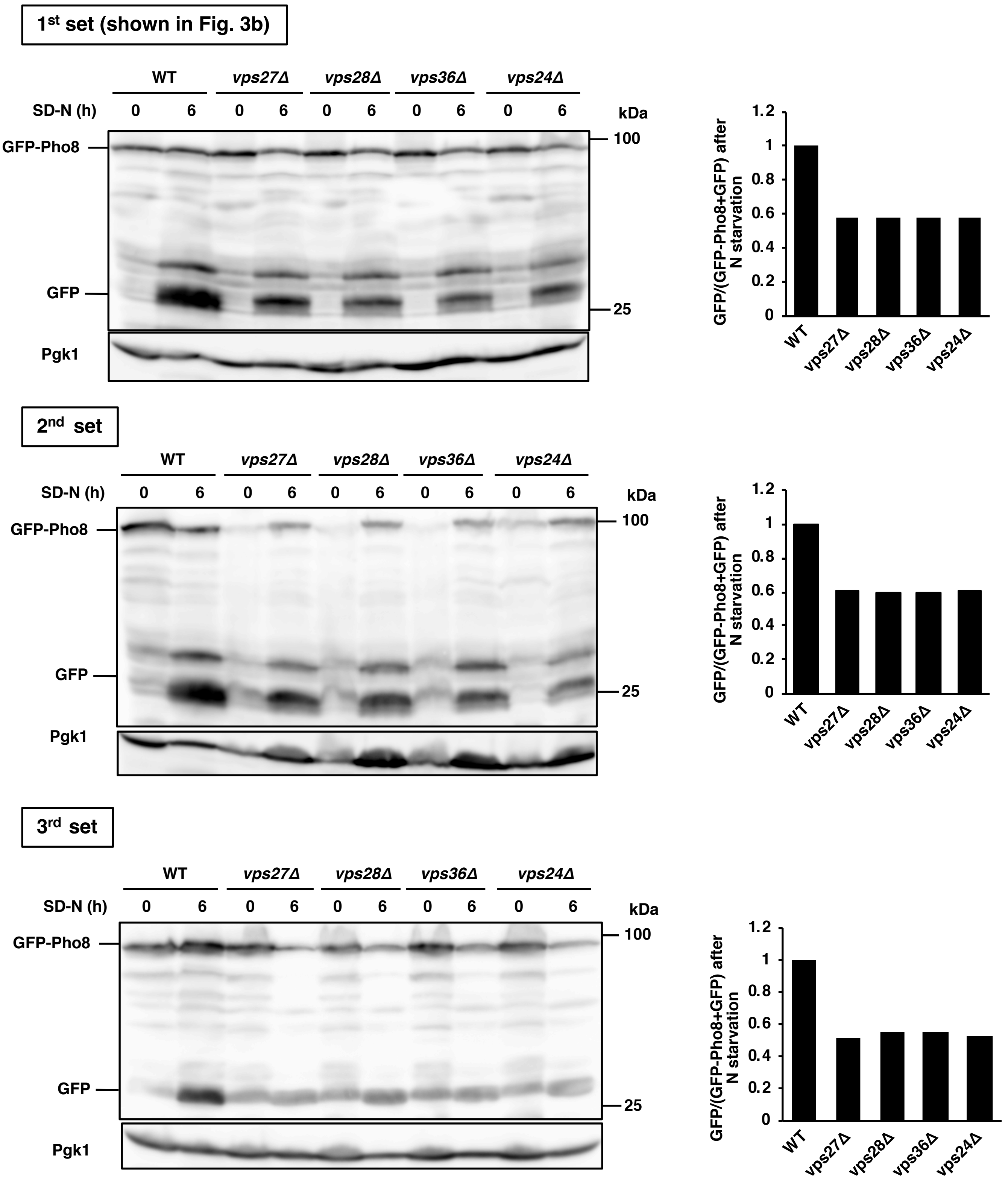

Fig. S4. Independent western blot images with quantifications (Related to Fig. 3b).

Fig. S5

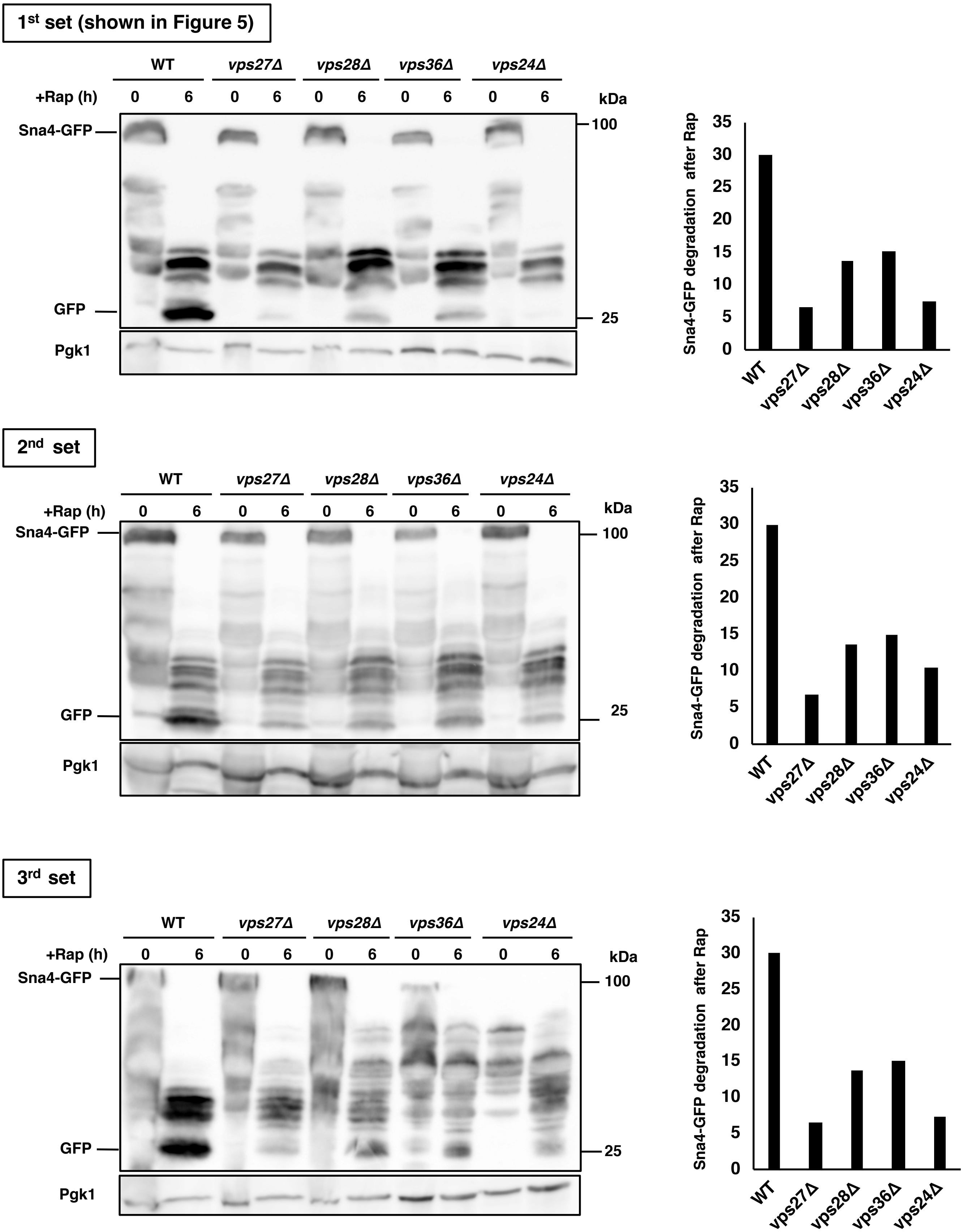

Fig. S5. Independent western blot images with quantifications (Related to Fig. 5).
