# Supplementary material for: ESCRT machinery plays a role in microautophagy in yeast
Source: BMC Mol Cell Biol. 2020 Oct 7;21:70. doi: 10.1186/s12860-020-00314-w (PMC7542719; doi:10.1186/s12860-020-00314-w)
Supplement: Supplementary file 2 — Additional file 2 Table S1. Yeast strains used in this study. [file 12860_2020_314_MOESM2_ESM.pdf]

Table S1. Yeast strains used in this study

Name	Description (source)
BY4741	*Mata leu2Δ ura3Δ0 his3-Δ1 met15Δ0* (lab stock)
SCU4337	BY4741 *vps24::kanMX* (EUROSCARF) [1]
SCU5456	BY4741 *vps27::kanMX* (EUROSCARF) [1]
SCU6187	BY4741 *vps28::kanMX* (EUROSCARF) [1]
SCU6188	BY4741 *vps36::kanMX* (EUROSCARF) [1]
SCU6206	BY4741 *YCK3-GFP::HIS3* (Invitrogen) [2]
SCU6207	BY4741 *NYV1-GFP::HIS3* (Invitrogen) [2]

References

1. Winzeler, E. A., Shoemaker, D. D., Astromoff, A., Liang, H., Anderson, K., Andre, B., Bangham, R., Benito, R., Boeke, J. D., Bussey, H., Chu, A. M., Connelly, C., Davis, K., Dietrich, F., Dow, S. W., El Bakkoury, M., Foury, F., Friend, S. H., Gentalen, E., Giaever, G., Hegemann, J. H., Jones, T., Laub, M., Liao, H., Liebundguth, N., Lockhart, D. J., Lucau-Danila, A., Lussier, M., M'Rabet, N., Menard, P., Mittmann, M., Pai, C., Rebischung, C., Revuelta, J. L., Riles, L., Roberts, C. J., Ross-MacDonald, P., Scherens, B., Snyder, M., Sookhai-Mahadeo, S., Storms, R. K., Veronneau, S., Voet, M., Volckaert, G., Ward, T. R., Wysocki, R., Yen, G. S., Yu, K., Zimmermann, K., Philippsen, P., Johnston, M. & Davis, R. W. (1999) Functional characterization of the S. cerevisiae genome by gene deletion and parallel analysis, *Science*. **285**, 901-6.
2. Huh, W. K., Falvo, J. V., Gerke, L. C., Carroll, A. S., Howson, R. W., Weissman, J. S. & O'Shea, E. K. (2003) Global analysis of protein localization in budding yeast, *Nature*. **425**, 686-91.
