# Supplementary material for: ESCRT machinery plays a role in microautophagy in yeast
Source: BMC Mol Cell Biol. 2020 Oct 7;21:70. doi: 10.1186/s12860-020-00314-w (PMC7542719; doi:10.1186/s12860-020-00314-w)
Supplement: Supplementary file 3 — Additional file 3 Table S2. Plasmids used in this study. [file 12860_2020_314_MOESM3_ESM.pdf]

Table S2. Plasmids used in this study

Plasmid	Description (source)
pSCU2366	pRS413 *MET25-GFP-PHO8 CEN HIS3* [1]
pSCU2425	pRS413 *VPH1-GFP CEN HIS3* [2]
pSCU2475	pRS413 *SNA4-GFP CEN HIS3* (this study)

References

1. Rahman, M. A., Terasawa, M., Mostofa, M. G. & Ushimaru, T. (2018) The TORC1-Nem1/Spo7-Pah1/lipin axis regulates microautophagy induction in budding yeast, *Biochem Biophys Res Commun*. **504**, 505-512.
2. Sharmin, T., Morshed, S. & Ushimaru, T. (2020) PP2A promotes ESCRT-0 complex formation on vacuolar membranes and microautophagy induction after TORC1 inactivation, *Biochem Biophys Res Commun*. **524**, 614-620.
